# Substrate type and palaeodepth do not affect the Middle Jurassic taxonomic diversity of crinoids

**DOI:** 10.7717/peerj.12017

**Published:** 2021-09-13

**Authors:** Mariusz A. Salamon, Anna Feldman-Olszewska, Sreepat Jain, Bruno B.M. Ferré, Karolina Paszcza, Bartosz J. Płachno

**Affiliations:** 1 Faculty of Natural Science, University of Silesia in Katowice, Sosnowiec, Poland; 2 Polish Geological Institute - National Research Institute, Warszawa, Poland; 3 Department of Applied Geology, School of Applied Natural Sciences, Adama Science and Technology University, Adama, Ethiopia; 4 Saint Étienne du Rouvray, France; 5 Faculty of Natural Sciences, University of Silesie in Katowice, Sosnowiec, Poland; 6 Faculty of Biology, Institute of Botany, Jagiellonian University in Kraków, Kraków, Poland

**Keywords:** Echinoderms, Crinoids, Jurassic, Bathonian, Callovian, Poland, Boreholes

## Abstract

Crinoids are largely considered as good indicators for determining environmental conditions. They are robust proxies for inferring changes in salinity and sedimentation rate and for inferring substrate type. Some crinoid groups (*e.g*., certain comatulids, cyrtocrinids, millericrinids) have a depth preference, thus, making them useful for palaeodepth estimation. The hypotheses that crinoid distribution is substrate-dependent (rock type) or palaeodepth-dependent is tested here based on (a) archival Bathonian-Callovian (Middle Jurassic) crinoid occurrences from Poland and (b) newer finds from five boreholes from eastern Poland. Qualitative data suggests that isocrinids and cyclocrinids occur in both carbonate and siliciclastic rocks. The cyrtocrinids and roveacrinids occur within carbonate rocks, whereas the comatulids are exclusive to siliciclastics. In terms of palaeodepth, most crinoid groups dominate in shallow environments with the sole exception of cyrtocrinids, that are ubiquitous and occur in both shallow (near shore and shallow marine) and slightly deeper (deeper sublittoral to open shelf) settings. The occurrences of the cosmopolitan taxa, Chariocrinus andreae and Balanocrinus subteres (isocrinids), is independent of both substrate type and palaeodepth. Quantitative analyses (Analysis Of Variance; ANOVA) based on substrate type, *i.e.*, substrate-dependency (claystones, sandstones and limestones), and palaeodepth *i.e*., palaeodepth-dependency (near shore, shallow-marine, mid-ramp and offshore), corroborate qualitative results. Statistical analysis suggest that the distribution of crinoids shows a strong substrate-dependency but not for palaeodepth, although very weak significance (low p value) is noted for near shore and shallow marine settings and crinoid distribution.

## Introduction

The Middle Jurassic (especially Bajocian and Callovian) strata of the epicratonic Poland (Central European province = Submediterranean province) are well-known for their excellently preserved and strongly diversified fossil fauna and flora (*e.g.*, Kopik, 1974; Kopik, 1976; Kopik, 1979a; Kopik, 1979b; Kopik, 1998; Kopik, 2006; Zatoń, Marynowski & Bzowska, 2006a; Zatoń et al., 2006b). These occur within clays and carbonate concretions and are represented by ammonites, gastropods, scaphopods, bivalves, belemnites, brachiopods, bryozoans, echinoids, asteroids, ophiuroids, wood fragments, and many others (for details see Matyja & Wierzbowski, 2000; Matyja & Wierzbowski, 2003; Gedl et al., 2003; Kaim, 2004; Zatoń & Marynowski, 2004; Zatoń & Marynowski, 2006; Zatoń, Marynowski & Bzowska, 2006a; Zatoń et al., 2006b; Zatoń, 2010a; Zatoń, 2010b; Zatoń, Wilson & Zavar, 2011). Crinoids, the group under study, are also diverse and abundant (more so in the boreholes of eastern Poland), and often form encrinites (a grain-supported bioclastic sedimentary rock) in which all or most of the grains are crinoid ossicles (*e.g.*, Salamon & Feldman-Olszewska, 2018). Despite such rich diversity and abundance, the crinoid data from the Middle Jurassic of Poland has largely been relegated to either brief mentions of species occurrences or of citing crinoid-yielding localities (see Salamon & Feldman-Olszewska, 2018 and literature cited therein).

**Figure 1:**
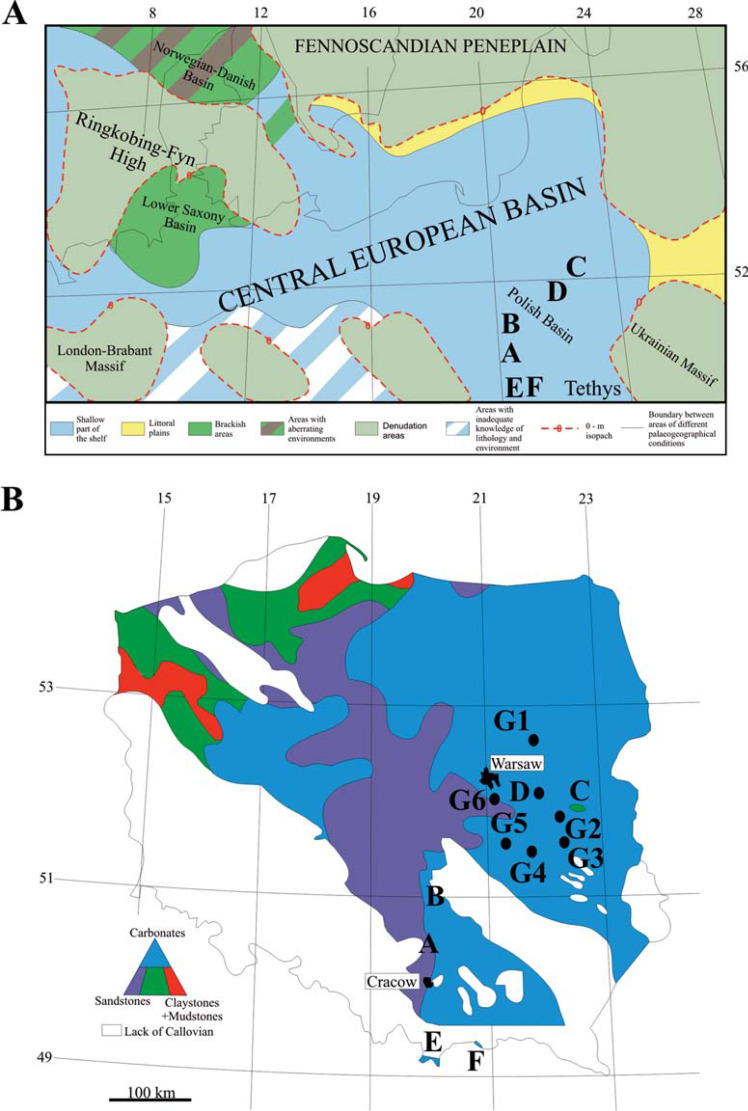
Location maps. (A) Palaeogeographic map of central Europe during the Middle Jurassic (modified after Šimkevičius, Ahlberg & Grigelis, 2003). (A) Bathonian and Callovian exposures of the southern part of the Polish Jura; (B) Bathonian and Callovian exposures of the southern margin of the Holy Cross Mountains; (C) Callovian exposure of the glacial drift in Łuków; (G1-G6) location of the Bathonian-Callovian boreholes described in this constribution [Tłuszcz IG1 (G1), Siedliska IG1 (G2), Kock IG2 (G3), Żyrzyn IG 1 (G4), Maciejowice IG1 (G5), Magnuszew IG1 (G6)], (D) location of borehole Żebrak IG1 (details in Salamon & Feldman-Olszewska, 2018); (A–D) Central European province; (E) Bathonian and Callovian exposures of the Tatra Mountains; (F) Bathonian and Callovian exposures of the Pieniny Klippen Belt; (E, F) Tethyan province. (B) Map of Callovian lithofacies noted in Poland (modified after Dayczak-Calikowska & Moryc, 1988).

The present Middle Jurassic study, based on new data from five eastern Poland boreholes (see Fig. 1 and Table 1) and previously published crinoid records, has twin objectives: (a) to standardize crinoid taxonomic disparity with added remarks on systematic issues and previous collections, and (b) to test two hypotheses using this standardized dataset. These include: (1) Do crinoids occur equally and frequently in both terrigenous and carbonate facies (test for substrate-dependency)? (2) Do crinoids occur equally and frequently in both deep and shallow water environments (test for palaeodepth-dependency)? Besides, qualitative analysis, the analysis of variance (ANOVA) is used to quantitatively test these two hypotheses.

During the Bathonian-Callovian duration, the Polish Basin was part of the epicratonic Central European Basin that separated the Fennoscandian plains on the north from the Western Tethys to the south. Only the extreme southern part of Poland (Tatra Mountains and Pieniny Klippen Belt) was part of the Tethyan realm (see Fig. 1). These two realms display distinct provincial characteristics (as evident from the distribution of ammonites; see Hallam, 1975; Brand, 1986), but this provincialism is not noted for crinoids. Hence, this study, also analyses crinoid taxonomic diversity trends between these two palaeobiogeographic provinces, *i.e.,* Tethyan and Central European.

### Previous records

The Middle Jurassic (Callovian) crinoids of the epicratonic basins of Poland have been recorded from the Polish Jura Chain (‘A’ on Fig. 1) and the Mesozoic margin of the Holy Cross Mountains (‘B’ on Fig. 1). Early records only mentioned crinoid localities (*e.g.*, Wójcik, 1910; Makowski, 1952; Różycki, 1953). Later, Dayczak-Calikowska (1980) listed several taxa but did not mention the exact locations from where the crinoid specimens were collected or where they were kept (sample repository). Furthermore, neither description, nor illustration, or even basic taxonomic group assignment was provided; most, however, belong to balanocrinids. Radwańska & Radwański (2003) from northern Poland recorded Callovian columnals of *Cyclocrinus macrocephalus* and Oxfordian cyclocrinid of *Cyclocrinus couiavianus*. Salamon & Zatoń (2006) from the Callovian marly limestones of the Zalas Quarry in southern Poland (‘A’ in Fig. 1; see also Table 2) erected a new species of balanocrinid (*Balanocrinus hessi*); this was later synonymized with *B. pentagonalis* by Krajewski, Olchowy & Salamon (2019). Salamon & Gorzelak (2007), from the same locality (Zalas Quarry in southern Poland; ‘A’ in Fig. 1) described few isolated remains of indeterminable cyrtocrinids associated with the cyrtocrinid cup of *Dolichocrinus* cf. *aberrans* (see Table 2). Salamon & Zatoń (2007) from a collection of ∼1,500 remains of columnals, pluricolumnals, brachials and cups, illustrated 11 crinoid taxa, including some isocrinids, comatulids, millecrinids and cyclocrinids (see Table 2). These specimens came from three Bajocian, four Bathonian and two Callovian localities of the southern part of the Polish Jura Chain and the Mesozoic margin of the Holy Cross Mountains (see Salamon & Zatoń, 2007). Current re-examination of this material led to the assignment of the columnals described as *Millericrinina* to the cyrtocrinids, Cyrtocrinida indet. (see Salamon & Feldman-Olszewska, 2018) (Table 2). Salamon (2008a) and Salamon (2008b) from two Callovian localities (Polish Jura Chain, ‘A’ in Fig. 1 and the “glacial drift” of Łuków in eastern Poland: ‘C’ in Fig. 1) recorded several crinoid taxa. The “glacial drift” assemblage of Łuków (‘C’ in Fig. 1) is dominated by isocrinids with associated comatulids. The carbonate rocks of the Polish Jura Chain yielded a rich assemblage of cyrtocrinids with dozens of complete individuals and a few isolated isocrinid remains. Salamon & Feldman-Olszewska (2018) from the Callovian crinoidal limestones of the Żebrak IG 1 borehole (eastern Poland; ‘D’ in Fig. 1) described a collection of five isocrinid taxa with some unidentifiable cyrtocrinids (see also Table 2). This collection contained a relatively small number of complete or nearly complete individuals, as a side-effect of the maceration process. Before the maceration process, the samples formed a typical encrinite and consisted largely of crinoids, as is also the case in the present study, yielding only fragmentary crinoids (see Table 2). Additionally, the Middle Jurassic (Bathonian-Callovian carbonates) exposures in southern Poland (Tethyan province, ‘E’ and ‘F’ in Fig. 1) have also yielded isocrinids, cyrtocrinids, roveacrinids, and cyclocrinids (see Głuchowski, 1987; see Table 2).

**Table 1:** Bathonian-Callovian crinoids collected in particular boreholes in eastern Poland.

Borehole	Depth (in m)	Age	Lithology	Determinable crinoid taxa
Kock IG 2	859.0	Callovian?	Yellowish grey, crinoidal limestone, packstone composed of echinoderm plates, bivalve shells, rare bryozoa fragments with ferruginous impregnations, ferruginous ooids, rounded quartz grains and sparite cement	Isocrinida indet. *Balanocrinus subteres*
Maciejowice IG 1	1,431.5	late Bathonian	Dark grey, medium-size sandstone	none
Magnuszew IG 1	1,505.7	late Callovian	Yellowish grey, crinoidal limestone, grainstone composed of echinoderm plates, echinoid spines, rounded quartz grains, sparite cement and iron oxides/hydroxides	Isocrinida indet. *Balanocrinus subteresPentacrinites dargniesi*
	1,507.3	late Callovian	Yellowish grey, crinoidal limestone with amonite and belemnite, grainstone composed of echinoderm plates, echinoid spines, bivalve shells, rounded quartz grains, sparite cement and iron oxides/hydroxides	Isocrinida indet. *Pentacrinites dargniesi*
	1,508.65	late Callovian	Grey crinoidal limestone, grainstone composed of echinoderm plates, echinoid spines, bivalve shells, bryozoa fragments, rare gastropoda, rounded quartz grains and sparite cement	Isocrinida indet. *Balanocrinus subteresPentacrinites dargniesi*
	1,522.4	middle-late Callovian	Yellow-brown, dolomitic crinoidal limestone with foraminifera, quartz grains and iron oxides/hydroxides	Isocrinida indet. *Isocrinus nicoletiBalanocrinus subteresBalanocrinus pentagonalisPentacrinites dargniesi*
Siedliska IG 1	824.65	early Callovian	Beige-grey, crinoidal limestone, grainstone composed of echinoderm plates, echinoid spines and bryozoa fragments frequently with ferruginous impregnations, crushed bivalve and brachiopods shells, gastropods, lithoclasts with iron hydroxides, rare ferruginous ooids, quartz grains, sparite or sparite-micrite cement and iron oxides/hydroxides	Isocrinida indet. *Pentacrinites dargniesi*
	828.25	early Callovian	Beige-grey, crinoidal limestonegrainstone composed of echinoderm plates, echinoid spines and bryozoa fragments frequently with ferruginous impregnations, crushed bivalve and brachiopods shells, gastropods, lithoclasts with iron hydroxides, rare ferruginous ooids, quartz grains, sparite or sparite-micrite cement and iron oxides/hydroxides	Isocrinida indet. *Balanocrinus subteresPentacrinites dargniesi*
	828.77	early Callovian	Beige-grey, crinoidal limestone grainstone composed of echinoderm plates, echinoid spines and bryozoa fragments frequently with ferruginous impregnations, crushed bivalve and brachiopods shells, gastropods, lithoclasts with iron hydroxides, rare ferruginous ooids, quartz grains, sparite or sparite-micrite cement and iron oxides/hydroxides	Isocrinida indet. *Isocrinus nicoletiBalanocrinus subteresPentacrinites dargniesi* Cyrtocrinida indet.
	830.8	late Bathonian?	Yellow-brown, crinoidal limestone with abundant iron hydroxides, lithoclasts and bivalve shells	none
	837.65	late Bathonian?	Yellow crinoidal limestone, grainstone composed of echinoderm plates, echinoid spines and bryozoa fragments frequently with ferruginous impregnations, crushed bivalve and brachiopods shells, gastropods, lithoclasts with iron hydroxides, rare ferruginous ooids, quartz grains, sparite or sparite-micrite cement and iron oxides/hydroxides	Isocrinida indet. *Chariocrinus andreaeBalanocrinus subteresBalanocrinus pentagonalisPentacrinites dargniesi*
Tłuszcz IG 1	1,047.7	late Callovian?-early Oxfordian	Beige-grey, crinoidal limestone	none
	1,049.95	late Callovian	Cream crinoidal limestone, grainstone composed of echinoderm plates, echinoid spines and bryozoa fragments, bivalve shells, very rare foraminifera, sparite cement and iron oxides/hydroxides	Isocrinida indet. *Balanocrinus pentagonalisPentacrinites dargniesiPhyllocrinus* sp.
Żyrzyn IG 1	1,134.95	Callovian	Yellowish grey, crinoidal limestone, grainstone composed of echinoderm plates, bryozoa fragments, bivalve shells, quartz grains, sparite cement and iron oxides/hydroxides	Isocrinida indet. *Chariocrinus andreaePentacrinites dargniesi* Cyrtocrinida indet.
	1,137.7	late Bathonian?	Beige-grey, crinoidal limestone with cement and iron hydroxides	none
	1,147.9	late Bathonian	Beige-grey, crinoidal limestone, grainstone composed of echinoderm plates, echinoid spines, bryozoa fragments, bivalve and brachiopods shells, quartz grains, sparite cement and iron oxides/hydroxides	Isocrinida indet. *Isocrinus nicoletiChariocrinus andreaeBalanocrinus subteresPentacrinites dargniesi* Cyrtocrinida indet.
	1,155.4	late Bathonian	Yellow-brown, crinoidal limestone, grainstone composed of echinoderm plates, bryozoa fragments, bivalve and brachiopods shells, sparite cement and iron oxides/hydroxides	Isocrinida indet. *Isocrinus nicoletiBalanocrinus pentagonalisPentacrinites dargniesi*

**Table 2:** Bathonian and Callovian crinoid list recorded in different areas of Poland.

Location area (see also details in Fig. 1 and its caption)	Age	Number of crinoid taxa	List of crinoids
C –glacial drift in Łuków; Central European province	Callovian	10, including: 8 isocrinids and 2 comatulids	**Isocrinids:***Chariocrinus andreaeBalanocrinus berchteniBalanocrinus pentagonalisBalanocrinus subteresIsocrinus* sp. *Isocrinus nicoletiIsocrinus pendulusPentacrinites* cf. *dargniesi***Comatulids:** Paracomatulidae sp. et gen. indet. *Palaeocomaster* sp.
G1-G6 –boreholes in eastern Poland (Kock IG 2, Maciejowice IG 1, Magnuszew IG 1, Siedliska IG 1, Tłuszcz IG 1, Żyrzyn IG 1); Central European province	latest Bathonian- Callovian	8, including: 6 isocrinids and 2 cyrtocrinids	**Isocrinids:** Isocrinida indet. *Chariocrinus andreaeBalanocrinussubteresBalanocrinus pentagonalisIsocrinus nicoletiPentacrinites dargniesi***Cyrtocrinids:** Cyrtocrinida indet. *Phyllocrinus* sp.
D –Żebrak IG 1; Central European province	Callovian	6, including: 5 isocrinids and 1 cyrtocrinid	**Isocrinids:***Chariocrinus andreaeBalanocrinus* cf. *subteresIsocrinus* sp. *Isocrinus* cf. *nicoletiPentacrinites* sp. **Cyrtocrinids:** Cyrtocrinida indet.
B –southern margin of the Holy Cross Mountains; Central European province	Bathonian	3, including: 2 isocrinids and 1 comatulid	**Isocrinids:** *Chariocrinus andreaeBalanocrinus berchteni* **Comatulids:** *Paracomatula helvetica*
B –southern margin of the Holy Cross Mountains; Central European province	Callovian	2, including: 1 isocrinid and 1 cyclocrinid	**Isocrinids:***Chariocrinus andreae***Cyclocrinids** (order uncertain according to Hess & Messing (2011)): *Cyclocrinus macrocephalus*
A –southern part of the Polish Jura; Central European province	Bathonian	6, including: 3 isocrinids, 2 comatulids and 1 cyrtocrinid	**Isocrinids:***Chariocrinus andreaeBalanocrinus berchteniIsocrinus bajociensis***Comatulids:***Paracomatula helveticaPalaeocomaster* sp. **Cyrtocrinids:** Cyrtocrinida indet. (=Millericrinina in Salamon & Zatoń (2007))
A –southern part of the Polish Jura; Central European province	Callovian	15, including: 3 isocrinids, 11 cyrtocrinids and 1 cyclocrinid	**Isocrinids:***Chariocrinus andreaeBalanocrinus subteresBalanocrinus pentagonalis* (=*Balanocrinus hessi* in Salamon & Zatoń (2006)) **Cyrtocrinids:** Cyrtocrinida indet. (=Millericrinina in Salamon & Zatoń (2007)) *Phyllocrinus* sp. *Phyllocrinus stellarisPhyllocrinus belbekensisTetracrinus moniliformisSclerocrinus* sp. *Cyrtocrinus* sp. *Pilocrinus moussoniDolichocrinus aberransFischericrinus ausichiLonchocrinus dumortieri***Cyclocrinids**: *Cyclocrinus macrocephalus*
E –Tatra Mountains; Tethyan province	Bathonian	8, including: 6 isocrinids, 1 cyrtocrinid and 1 cyclocrinid	**Isocrinids:***Isocrinus* sp. *Isocrinus bathonicusIsocrinus nicoletiIsocrinus pendulusBalanocrinus* sp. *Pentacrinites dargniesi***Cyrtocrinids:** Cyrtocrinida indet. **Cyclocrinids:***Cyclocrinus rugosus*
E –Tatra Mountains; Tethyan province	Callovian	3, including: 1 isocrinid, 1 cyrtocrinid and 1 roveacrinid	**Isocrinids:***Isocrinus* sp. **Cyrtocrinids:** Cyrtocrinida indet. **Roveacrinids:***Saccocoma* sp.
F –Pieniny Klippen Belt; Tethyan province	Bathonian	16, including: 10 isocrinids, 5 cyrtocrinids and 1 roveacrinid	**Isocrinids:***Chariocrinus andreaeIsocrinus* sp. *Isocrinus bathonicusIsocrinus nicoletiIsocrinus pendulusBalanocrinus* sp. *Balanocrinus berchteniBalanocrinus pentagonalisBalanocrinus subteresPentacrinites dargniesi***Cyrtocrinids:** Cyrtocrinida indet. *Plicatocrinus tetragonusLonchocrinus dumortieriRemisovicrinus* sp. *Dolichocrinus* sp. *aberrans***Roveacrinids:***Saccocoma* sp.
F –Pieniny Klippen Belt; Tethyan province	Callovian	14, including: 2 isocrinids, 9 cyrtocrinids, 1 roveacrinid, and 2 cyclocrinids	**Isocrinids:***Balanocrinus subteresBalanocrinus pentagonalis***Cyrtocrinids:** Cyrtocrinida indet. *Lonchocrinus dumortieriRemisovicrinus* sp. *Dolichocrinus* cf. *aberransEudesicrinus* sp. *Pilocrinus moussoniGammarocrinites* sp. *Sclerocrinus compressusPhyllocrinus* sp. **Roveacrinids:***Saccocoma* sp. **Cyclocrinids:***Cyclocrinus* sp. *Cyclocrinus rugosus*

## Material and Methods

**Figure 2:**
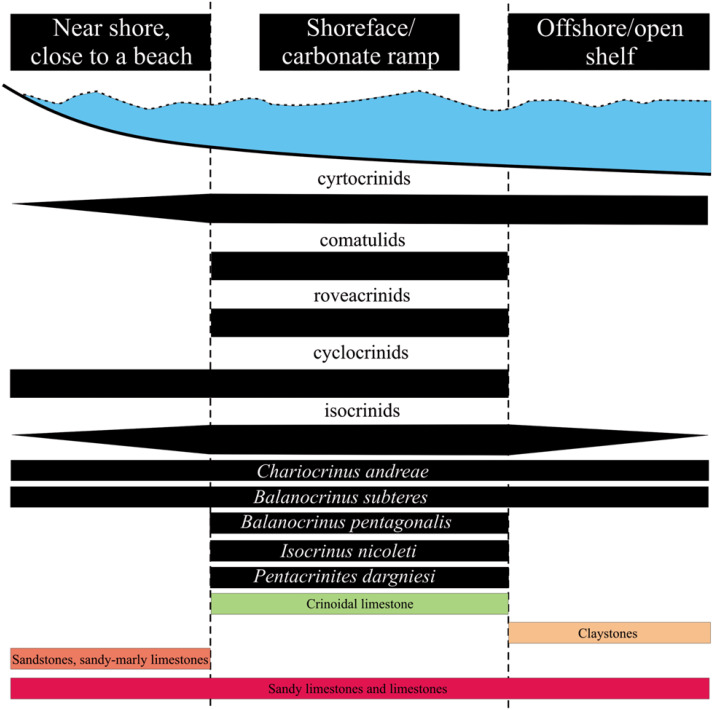
Model showing distribution of selected Callovian crinoids.

The archival cores (72 core samples) drilled in eastern Poland (G1–G6 on Figs. 1 and 2) were investigated for crinoids. These cores are stored in the Polish Geological Institute National Research Institute, Warsaw (Poland). The samples selected for the maceration process were selected from the following boreholes at respective depths: (1) Kock IG 2 (core depth: 859.0 m; age: Callovian?); (2) Maciejowice IG 1 (core depth: 1,431.5 m; age: late Bathonian); (3) Magnuszew IG 1 (core depths: 1,505.7 m, 1,507.3 m, 1,508.65 m, and 1,522.4 m; age: middle and late Callovian respectively); (4) Siedliska IG 1 (core depths: 824.65 m, 828.25 m, 828.77 m, 830.8 m, and 837.65 m; age: late Bathonian?-early Callovian); (5) Tłuszcz IG 1 (core depth: 1,047.7 m and 1,049.95 m; age: late Callovian?-early Oxfordian); (6) Żyrzyn IG 1 (core depths: 1,134.95 m, 1,137.7 m, 1,147.9 m, and 1,155.4 m; age: late Bathonian-Callovian) (for a summary see Table 2).

The present paper also includes a large collection of Callovian crinoids from Poland collected by Mariusz Salamon and Bartosz Płachno (2006-present) and others, mentioned in the *Acknowledgements* section.

The first step for the current analysis consisted of examining the drill cores under a binocular microscope for crinoids. Thereafter, the carbonate samples were soaked with Glauber’s salt. Based on sample cohesion, these were then successively boiled and freezed. The residue was then washed under running tap water and sieved (mesh sizes: Ø1.0, 0.315 and 0.1 mm, respectively). The final step consisted of drying the screened residue at 170–180 °C (following the methodology outlined by Krajewski, Olchowy & Salamon, 2019).

Both the sandstone sample Maciejowice IG 1 (core depth: 1,431.5 m; age: late Bathonian) and the carbonate sample Magnuszew IG 1 (core depth: 1,505.7 m; age: late Callovian) were naturally macerated and left outside for 30 days (from January 22nd to February 21st, 2021). As a result of this natural maceration, the samples were partially disintegrated. In addition to this, several thin sections (TS) and polished slabs (PS) were also made. All crinoids were hand-picked from the maceration residue and photographed using a Canon Eos 350D digital camera, LeicaWildM10 coupled with a NikCamPro1 microscope and a Scanning Electron Microscope Philips XL-20. All specimens are housed in the Institute of Earth Sciences of the University of Silesia in Katowice, Poland, and catalogued under registration number GIUS 8-3734. Other Middle Jurassic specimens used in the current study are also housed at the Institute of Earth Sciences of the University of Silesia in Katowice, Poland, and catalogued under: GIUS 8-2510, 8-2569, 8-2571, 8-3460Cr, 8-3466, 8-3678/1-6, 8-3734.

### Statistical methods

The taxonomically standardized dataset was subjected to Analysis Of Variance (ANOVA). To check for substrate-dependency (Hypothesis 1), the dataset was categorized under claystones, sandstones and limestones (the former two represent siliciclastics, whereas the latter, carbonates). For palaeodepth-dependency (Hypothesis 2), the dataset was categorized under near shore, shallow-marine, mid-ramp and offshore categories. For pairwise comparisons, the Tukey’s HSD (honestly significant difference) test was applied.

### Taxonomic standardisation

The cyrtocrinids are identified at the specific level based on their cups. In some cases, when the cups are fragmentary or some typical features are not visible, the samples were assigned to the generic level, only. Most of the disarticulated remains of cyrtocrinids (holdfasts, columnals, radials, basals, brachials, etc.) are assigned under Cyrtocrinida indet.

As for comatulid centrodorsals, where cups with basals and radials were not available for additional information, they were also classified at the generic level. The brachial with muscular articulation on the proximal side and syzygial one on the distal side are classified as Paracomatulidae sp. et gen. indet.

The isolated remains of isocrinids, consisting of columnals, pluricolumnals, cirrals, cup plates, and brachials, are classified at the specific level. If they were found in different levels, they are described as Isocrinida indet.

A complete individual of cyclocrinid has not been found so far. Despite this, the uniqueness of their remains (*e.g.*, large cylindrical columnals with peculiar tuberculate facets) allows us to identify them at the specific level (see detailed discussion in Radwańska & Radwański (2003). Głuchowski (1987) assigned some remains from the Pieniny Klippen Belt to *Cyclocrinus* sp. due to the incompleteness of columnals. Głuchowski (1987) classified all roveacrinids (saccocomids) as *Saccocoma* sp.; these were recorded either as isolated cup remains or observed in thin sections (TS).

**Table 3:** Distribution of Bathonian-Callovian crinoids recorded in Poland within different facies and setting.

**Abiotic factors**						1	5	2	4	2	2	2	2	2	3	1	2	3	3	3	2	1	1	1	1	1	1	1	1	2	1	1	1	1	1	1	1	1	4	1
**Lithology**	**Locality**	**Age**	**Energy level**	**Palaeodepth**	**Substrate**	Isocrinida indet.	Chariocrinus andreae	Balanocrinus sp.	Balanocrinus berchteni	Balanocrinus pentagonalis	Balanocrinus subteres	Isocrinus sp.	Isocrinus nicoleti	Isocrinus pendulus	Isocrinus bajociensis	Isocrinus bathonicus	Pentacrinites dargniesi	Paracomatulidae sp. et gen. indet.	Palaeocomaster sp.	Paracomatula helvetica	Cyrtocrinida indet.	Phyllocrinus sp.	Phyllocrinus stellaris	Phyllocrinus belbekensis	Tetracrinus moniliformis	Sclerocrinus sp.	Sclerocrinus compressus	Cyrtocrinus sp.	Pilocrinus moussoni	Dolichocrinus aberanns	Fischericrinus ausichi	Lonchocrinus dumortieri	Plicatocrinus tetragonus	Remisovicrinus sp.	Eudesicrinus sp.	Gammarocrinite s sp.	Saccocoma sp.	Cyclocrinus sp.	Cyclocrinus macrocephalus	Cyclocrinus rugosus
Claystones	B –southern margin of the Holy Cross Mountains	Bathonian	Low energy to moderately turbulent	Offshore below the wave base	Siliciclastics			20		200											3																
Claystones with associated carbonate concretions	C –glacial drift at Łuków	Callovian	Moderately turbulent to turbulent	Shallow-marine			104	17	101	5	33	87	17	7			11	5	1																	
Claystones with levels of siderites and carbonate concretions	A –southern part of the Polish Jura	Bathonian	Low energy to moderately turbulent	Offshore below the wave- base			60		800						4				1	5																
Organodetrital limy sandstones, sandy-marly limestones and marls	B –southern margin of the Holy Cross Mountains	Callovian	Turbulent	Near shore, close to a beach				10				10										4														24	
Sandstones	A –southern part of the Polish Jura	Callovian	Turbulent	Near shore, close to a beach				30										p									1										
Sandy-limestones and limestones	A –southern part of the Polish Jura	Callovian	Low energy to moderately turbulent	Deeper sublittoral with influence of storms to open shelf				35				45							1	13	4	6	3		3	8	3	ca. 10	7							6	
Crinoidal limestones	D –boreholes in eastern Poland (Kock IG 2, Maciejowice IG 1, Siedliska IG 1, Tłuszcz IG 1, Żyrzyn IG 1)	Uppermost Bathonian- Callovian	Turbulent	Shallow-marine	Carbonate		ca. 100	ca. 100			28	ca. 120		ca. 70				ca. 80				ca. 150	2														
Crinoidal limestones	D –Żebrak IG 1	Callovian	Turbulent	Shallow-marine				40				4	30	9				47				10															
Crinoidal limestones	E –Tatra Mountains	Bathonian	Turbulent	Shallow-marine					p				p	p	p		p	p				p															p
Crinoidal limestones	E –Tatra Mountains	Callovian	Turbulent	Shallow-marine									p					p																p			
Crinoidal limestones	F –Pieniny Klippen Belt	Bathonian	Turbulent	Shallow-marine				p	p	p	p	p	p	p	p		p	p				p					p		p	p	p			p			
Crinoidal limestones	F –Pieniny Klippen Belt	Callovian	Turbulent	Shallow-marine							p	p						p						p		p	p		p		p	p	p	p	p		p

Palaeodepth	Isocrinida indet.	Chariocrinus andreae	Balanocrinus sp.	Balanocrinus berchteni	Balanocrinus pentagonalis	Balanocrinus subteres	Isocrinus sp.	Isocrinus nicoleti	Isocrinus pendulus	Isocrinus bajociensis	Isocrinus bathonicus	Pentacrinites dargniesi	Paracomatulidae sp. et gen. indet.	Palaeocomast er sp.	Paracomatula helvetica	Cyrtocrinida indet.	Phyllocrinus sp.	Phyllocrinus stellaris	Phyllocrinus belbekensis	Tetracrinus moniliformis	Sclerocrinus sp.	Sclerocrinus compressus	Cyrtocrinus sp.	Pilocrinus moussoni	Dolichocrinus aberanns	Fischericrinus ausichi	Lonchocrinus dumortieri	Plicatocrinus tetragonus	Remisovicrinus sp.	Eudesicrinus sp.	Gammarocrini tes sp.	Saccocoma sp.	Cyclocrinus sp.	Cyclocrinus macrocephalus	Cyclocrinus rugosus
6		20		200											3														
6		104	17	101	5	33	87	17	7			11	5	1															
6		60		800						4				1	5														
6		10				10										4																		24	
6		10				10										4																		24	
6						30																													
7		35				45											1	13	4	6	3		3	8		ca. 10	7							6	
6	ca. 100	ca. 100			28	ca. 120		ca. 70				ca. 80				ca. 150	2																		
6		40				4	30	9				47				10																			
6			p				p	p	p		p	p				p																			p
6							p									p																p			
6		p	p	p	p	p	p	p	p		p	p				p									p		p	p	p			p			
6					p	p										p						p		p	p		p		p	p	p	p	p		p
6					p	p										p						p		p	p		p		p	p	p	p	p		p
6																																		

**Notes.**

1 Exclusively in carbonates (column) 2 Predominance in carbonates (column) 3 Exclusively in siliciclastics (column) 4 Predominance in siliciclastics (column) 5 Cosmopolitan (column) 6 Near shore and shallow marine (row) 7 Deep sublittoral with influence of storms to open shelf (row)

**Figure 3:**
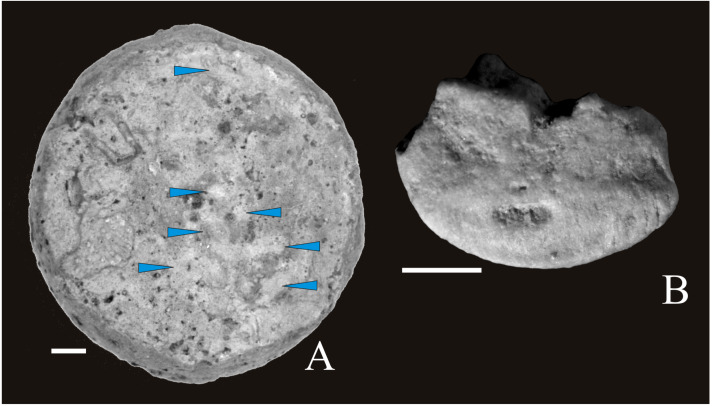
Cyclocrinid remains from southern Poland. (A) Columnal, articular facet. (B) Brachial plate. Scale bar equals one mm. Blue arrows mark granules covering the articular facet of columnal.

Thus, the dataset used in the present study is taxonomically standardised. The crinoids classified in the present study are given in Tables 1–4. A summary of all data with their respective inferred palaeodepth and species diversity (number of taxa) is given in Table 3 (see also Fig. 3).

**Table 4:** Distribution of Bathonian-Callovian crinoids recorded in Poland within different facies and settings.

**Species**	**Details**
Isocrinida indet.	Plenty of more or less complete columnals, brachials, and cirrals
*Isocrinus nicoleti* (Desor, 1845)	Plenty of more or less complete, isolated columnals
*Chariocrinus andreae* (Desor, 1845)	Plenty of more or less complete, isolated columnals
*Balanocrinus subteres* (Münster in Goldfuss, 1826)	Plenty of more or less complete, isolated columnals
*Balanocrinus pentagonalis* (Goldfuss, 1826-1844)	21 almost complete columnals
*Pentacrinites dargniesi* Terquem & Jourdy, 1869	Plenty of cirrals, 14 almost complete columnals
Cyrtocrinida indet.	Plenty of cup remains and partly preserved columnals and 27 nearly complete columnals
*Phyllocrinus* sp.	Two cups

## Results

The present study identified several crinoid remains of cups, columnals, cirrals and brachials from the 5 boreholes drilled in eastern Poland (Fig. 1). Only two pluricolumnals were recorded. The first one consists of three or four columnals observable on the surface of a rock fragment from the Magnuszew IG 1 core (depth: 1,505.7 m). The second one consists of two columnals and comes from the sample of Siedliska IG 1 (depth: 828.77 m). The other echinoderms observed on slab surfaces include numerous partly preserved cidaroid spines, cidaroid interambulacral plates and asteroid marginal plates. The echinoderm remains were also observed in thin sections and on the surface of polished slabs. In addition to these echinoderm remains, bivalves, gastropods and ostracods were observed both on the slab surfaces and in the macerated residues. In the late Bathonian sample Maciejowice IG 1, the remains of carbonized plant remains were also observed (for a summary of the above data see Table 4). In general, the Bathonian and Callovian crinoid assemblages are almost identical (see also Table 1). The other investigated Middle Jurassic crinoids were represented by numerous cups, cup remains, brachials, columnals, pluricolumnals, cirri, cirrals, and holdfasts (for details see Głuchowski, 1987; Salamon & Gorzelak, 2007; Salamon & Zatoń, 2007; Salamon, 2008a; Salamon, 2008b; Salamon, 2008c; Salamon & Feldman-Olszewska, 2018).

### State of preservation

The preservation of crinoids was examined only on the non-macerated surfaces of fresh core fragments. These were screened for signs of abrasion, bioerosion traces, chemical alteration of ossicle structure, evidence of epibionts and predation traces. Most of the crinoids display a high degree of disarticulation (almost 100%). However, they are all well preserved and do not show any traces of lateral surface abrasion. Excellent preservation and lack of abrasion are also noticed for columnal articular surfaces; the crenulae, petal floors, perilumens and lumens are complete. The two pluricolumnals also show no signs of abrasion. Additionally, the studied crinoid remains are very rarely bioeroded (bioerosion was only noticed in case of three columnals in the form of small and rounded holes). These may be ascribed to acrothoracican cirripedes, algae, fungi, polychaetes, sipunculans, or to sponge activities (for more details see Salamon & Zatoń, 2006; Zatoń, Villier & Salamon, 2007; Salamon & Gorzelak, 2010).

Donovan (1991) suggested that under normal oxygenated conditions, complete crinoid disarticulation takes place within two weeks. However, Brett, Moffat & Taylor (1997) argued that under certain circumstances, complete disarticulation may occur even after a year. In living comatulids, disarticulation starts just after death and by the end of the first week, only isolated calyx and a few arm fragments remain articulated (Ausich, 2001). Under high water temperatures and with increased physical disturbance, the disarticulation process significantly speeds up (see Ausich, 2001; Hess, 2006). Baumiller & Ausich (1992) noted that after 19 days, the isocrinid stem disarticulates into noditaxes and after 22 days, the column segments get disarticulated into isolated columnals. Gorzelak & Salamon (2013) noted that under high-energy conditions (constant transportation), and at room temperature, disarticulation of the crinoid skeleton is nearly complete after 17 days, where only a few articulated arms and cirral ossicles remain intact.

In the present study, the crinoid remains are preserved as cups, isolated columnals, brachials, and cups. Therefore, these remains can confidently be classified as taphonomic types 2 and 3 *sensu* Brett, Moffat & Taylor (1997). Type 2 includes isocrinids with some remains of cyrtocrinid that undergo rapid postmortem disarticulation, on account of weak sutures. Type 3 comprises of cyrtocrinid cups in which major portions of the skeleton are resistant to disarticulation.

Thus, for the present study, all the data suggest that the recorded crinoids are autochthonous with negligible or no post-mortem transportation. Possibly, after death, they remained for some time on the sea bottom, but were not subjected to significant transportation or reworking before burial, or they were shortly covered by sediment, as suggested by the lack of abrasion surfaces. Bearing this in mind we assume little time-averaging that might alter the palaeoecological and palaeodepth information. Additionally, no trace of chemical alteration, predation traces and epibionts were noticed, suggesting that the inferred palaeoecological and palaeodepth signals are primary.

### Statistical analyses

#### Substrate dependency (Hypothesis 1)

The ANOVA test (Table 5a) yielded statistically significant differences among the fauna of the different substrate types (claystones, sandstones and limestones) at *p* < .001. For pairwise comparisons, the Tukey’s HSD (honestly significant difference) test was applied (Table 5b). This yielded a significant difference between both claystones-sandstones and limestones but no significance difference between claystones and sandstones (both being siliciclastics; Table 5b).

**Table 5:** Analysis of variance (ANOVA). (A) ANOVA test between different substrate types (Claystones, Sandstones and Limestones). (B) Tukey’s HSD (honestly significant difference) test for pairwise comparisons. (C) ANOVA test between different palaeodepth parameters (Near shore, Shallow-marine, Mid-ramp and Offshore). (D) Tukey’s HSD (honestly significant difference) test for pairwise comparisons.

**a**				
***Source***	***SS***	***df***	***MS***	
Between-treatments	96	2	48	*F*= 8.57872
Within-treatments	335.7143	60	5.5952	
Total	431.7143	62		
**b**				
Pairwise comparisons			HSD_.05_= 1.7543 HSD_.01_= 2.2104	Q_.05_= 3.3987 Q_.01_= 4.2822
Claystones: Sandstones	M1 = 0.90 M2 = 0.33		0.57	Q = 1.11 (*p*= .71491)
Claystones: Limestones	M1 = 0.90 M3 = 3.19		2.29	Q = 4.43 (*p*= .00748)
Sandstones: Limestones	M2 = 0.33 M3 = 3.19		2.86	Q = 5.54 (*p*= .00068)
**c**				
***Source of variation***	***SS***	***df***	***MS***	
Between-treatments	28.9	3	9.6333	*F*= 3.32335
Within-treatments	220.3	76	2.8987	
Total	249.2	79		
**d**				
*Pairwise Comparisons*			HSD_.05_= 1.4143 HSD_.01_= 1.7333	Q_.05_= 3.7149 Q_.01_= 4.5530
T_1_:T_2_	M_1_= 0.35		1.65	Q = 4.33 (*p*= .01563)
	M_2_= 2.00			
T_1_:T_3_	M_1_= 0.35		0.5	Q = 1.31 (*p*= .78958)
	M_3_= 0.85			
T_1_:T_4_	M_1_= 0.35		0.85	Q = 2.23 (*p*= .39675)
	M_4_= 1.20			
T_2_:T_3_	M_2_= 2.00		1.15	Q = 3.02 (*p*= .15112)
	M_3_= 0.85			
T_2_:T_4_	M_2_= 2.00		0.8	Q = 2.10 (*p*= .45097)
	M_4_= 1.20			
T_3_:T_4_	M_3_= 0.85		0.35	Q = 0.92 (*p*= .91521)
	M_4_= 1.20			

#### Palaeodepth-dependency (Hypothesis 2)

The comparison between Palaeodepth parameters (near shore, shallow-marine, mid-ramp and offshore) did not yield any statistically significant results (Table 5c). The pairwise comparisons, Tukey’s HSD test (Table 5d) retained a statistically significant difference only between the first two variables (Near shore and Shallow marine); although, the *p* value is very low (*p* > 0.01).

## Discussion

### Depositional environment of the eastern part of the epicontinental Polish Basin

Upper Bathonian and Callovian of the eastern part of Polish Basin are represented by organodetritic, mostly crinoidal limestones with abundant limonite. There are grainstones and packstones which are dominated by echinoderm plates, less frequently appear echinoid spines, bryozoans, bivalves and brachiopods, occasionally gastropods, foraminifera, ammonites and belemnites are present. Quartz grains are frequently an admixture. In places also ferruginous ooids are present. Sedimentary environment is interpreted as a mid-ramp located between normal and storm wave base (Feldman-Olszewska, 2018).

### Depositional environment of the Łuków glacial draft

The Łuków glacial drift exposed in eastern Poland (‘C’ in Fig. 1), is a 30 m-thick Callovian unit containing black clays with carbonate concretions (see also Mizerski & Szamałek, 1985), deposited in an offshore setting (*e.g.*, Olempska & Błaszyk, 2001; Kaim, 2004). However, recently, based on the presence of a high diversity asteroid assemblage, a varied environment (ranging from coastal to deep-shelf) was proposed (Villier, 2008). This unusually high crinoid diversity is likely a reflection of ossicles being transported by currents from shallow- to deep-water offshore environments (Villier, 2008). However, according to Salamon (2008a), based on the ecology of crinoids, the Łuków clays was deposited in a relatively shallow environment and contrary to asteroids, they were not transported. Based on the presence of macro- and micro-remains of land flora, a nearshore environment was also inferred (see also Brand, 1986; Marynowski et al., 2008). Similarly Gedl (2008) using dinoflagellate cysts concluded that these originated presumably from shallow marine environments. Sedimentological investigations of the Aalenian-Bathonian (Middle Jurassic) claystones and shales from central Poland indicate that their sedimentation took place in offshore (below storm wave base) and transition (between normal and storm wave base) zones of the epeiric sea (see Feldman-Olszewska, 2007; Feldman-Olszewska, 2008; Feldman-Olszewska, 2012a; Feldman-Olszewska, 2012b; Pieńkowski et al., 2008). Water depth corresponds to the *Cruziana* ichnofacies but shallower than the *Zoophycus* ichnofacies (see Knaust & Bromley, 2012). The same depositional environment is expected for the Callovian clays from Łuków.

### Problems with isolated isocrinid classification

The isocrinids are a dominant crinoid group in the Middle Jurassic sediments of Poland. However, their correct classification into families and genera is very complicated. The ideal situation is when complete crinoid findings are available (*e.g.*, Simms, 1989; Hess & Messing, 2011), but these are rare due to the unique structure of their skeletons (see *State of preservation*). There are of course also isocrinids that have a unique structure of columnals and it is easier to identify them from others. A classic example is that of *Balanocrinus subteres* or *B. pentagonalis* (see Klikushin, 1992; Hess, 2014a; Hess, 2014b; Krajewski, Olchowy & Felisiak, 2016; Krajewski, Olchowy & Salamon, 2019; Krajewski, Ferré & Salamon, 2020). Hess (2014a) and Hess (2014b) stated that the columnals of *B. subteres* could be associated with its isolated calyx elements. Likewise, with remains of *Pentacrinites dargniesi* or *Isocrinus nicoleti* display a unique shape and ornamentation of their articular facets, as also *P. dargniesi* with its characteristic, ellipsoidal cirrals.

However, there is an issue with non-balanocrinid isocrinids, that have columnals of a similar morphology, and were erected on the basis of isolated skeletal remains. There are many such forms in the Middle and Upper Jurassic strata of Poland. According to De Loriol (1877-1879), the most similar taxon to *Isocrinus amblyscalaris* is *I*. *munieri*. But there are some noticeable differences between them, also *i.e.,* the height of columnals and the ornamentation of the latera. Hess (2014a) added that *Pentacrinus pellati* with low columnals alternating with higher ones, and ridges occurring on its latera, is also a very similar form. He concluded that both species are of middle Oxfordian age and therefore may be conspecific. In turn, Bather (1898) suggested that *I. amblyscalaris* currently from the Oxfordian and Kimmeridgian of Poland is a synonym of *I. pendulus*. Hess (1975) added that both these species originated from the same middle Oxfordian strata (Bärschwil Fm, NW Switzerland). On the other hand, Klikushin (1992) stated that there is a series of morphological differences between these two species that supersedes Bather’s (1898) opinion. The latter author, elsewhere in his monograph, however, proposed to consider *I. pendulus*, Oxfordian *I. amblyscalaris*, and Callovian-Oxfordian *I. oxyscalaris* as junior synonyms of the Oxfordian *I. desori*. Krajewski, Olchowy & Salamon (2019) differed from this view and emphasized that there are some differences in the facet morphology of the mentioned taxa. Apart from the differences in the articular facets (*i.e.,* the presence of deep furrows between petal floors), the shape of columnals may also be different (compare Hess, 1975, p. 55, text-fig. 8, pl. 19, fig. 4; Klikushin, 1992, pl. 11, fig. 1–7 *vs.* pl. 13, figs. 8–10; Radwańska, 2005, fig. 8/4; Salamon, 2009, fig. 2H).

Taking into account all of the above, it is difficult to say unequivocally whether there are one or more biological species within the Middle/Upper Jurassic strata of Poland. Therefore, such forms, whose taxonomic affiliation raises serious doubts (*Balanocrinus berchteni*, *Isocrinus bajocensis, I. bathonicus*, and *I. pendulus*), must be evaluated with great caution in any analysis.

### Taxonomic status of cyclocrinids

The cyclocrinids (Cyclocrinidae) were classified by Biese (1935-37) as millericrinids (Millericrinida, Apiocrinitidae). Sieverts-Doreck (Ubaghs, 1953) and later Hess (1975) placed the cyclocrinids within cyrtocrinids (Cyrtocrinida). According to Radwańska & Radwański (2003) cyclocrinid columnals are elements of radical cirrals and therefore belong to bourgueticrinids (Bourgueticrinida). Following Hess (2008), the ordinal position of *Cyclocrinus* and of the family Cyclocrinidae is left in the open nomenclature though the form has some resemblance to the Early Jurassic millericrinid genus *Amaltheocrinus*. Hess & Messing (2011) placed *Cyclocrinus* within uncertain order pending proper classification of its cup plates that still remain unknown.

One of the previously investigated Callovian sections (the abandoned Wysoka Quarry placed within the Polish Jura Chain; ’A’ in Fig. 1), exposes sandy limestones where numerous cyclocrinid columnals were collected from a narrow level. Remains of other crinoids were not found there. Along with these columnals, several “millericrinid”-like brachial (see Fig. 3) plates were found, which probably belong to the same crinoid as the columnals. Brachials display a proximal synostosis and a muscular distal side, sometimes with a pinnule socket. Both facets are parallel. This seems to confirm Hess’s (2008) idea of linking cyclocrinids to millericrinids. On the other hand, the “bourgueticrinid”-like, strongly abraded cup was found by us in the Czerwieniec locality by one of the authors (BJP). This cup is very similar in shape and size to bourgueticrinid cup that may suggest that cyclocrinids really belong to bourgueticrinids as suggested by Radwańska & Radwański (2003).

### Cyclocrinids from Poland

Wójcik (1910) first listed the occurrence of *Cyclocrinus macrocephalus* in Poland and the occurrence of columnals in the sands of Filipowice, Polish Jura Chain (‘A’ in Fig. 1). Głuchowski (1987) noted the occurrence of *Cyclocrinus rugosus* and *Cyclocrinus* sp. within the Bathonian and/or Callovian crinoidal limestones of the Tatra Mountains and the Pieniny Klippen Belt (‘E’ and ‘F’ in Fig. 1). Radwańska & Radwański (2003) questioned this assignment, claiming that the sketches and photographs are not of sufficient quality, specimens are poorly preserved, and that the ‘cyclocrind’ were small-sized. Re-examinations of Głuchowski specimens (1987, fig. 13/1, 13/4 and pl. 1, fig. 1–6), stored at the Institute of Earth Sciences of the University of Silesia in Katowice, Poland, prove that they are significantly smaller than the columnals collected from other areas. Additionally, they are cylindrical, low and with a slightly convex latera, suggesting that they may well belong to cyclocrinids. Therefore, the original designations of Głuchowski (1987) are currently upheld. Moreover, Radwańska & Radwański (2003) recognized four cyclocrinid species within the Middle and Upper Jurassic strata of Poland, namely: *Cyclocrinus rugosus* from the Bajocian, *C. macrocephalus* from the Callovian, *C*. *areolatus* from the Oxfordian, and *C. couiavianus* from the upper Oxfordian. They stated that all mentioned taxa (with the exception of *C. couiavianus*) lie within the variability of *C. rugosus* (Radwańska & Radwański, 2003)*.* Though describing columnals with articular surfaces covered by numerous and irregular tubercles, Salamon & Zatoń (2007) included these remains under *C. macrocephalus*. Re-observation of columnals coming from the same Callovian levels of the Polish Jura Chain, both ornamented by and devoid of tubercles, confirm the suggestion of Radwańska & Radwański (2003) that all these cyclocrinids actually belong to a single species, *Cyclocrinus rugosus*.

### Distribution of crinoids within particular facies. Does it really work?

Hans Hess in several of his papers pointed out that the occurrence of certain species of crinoids may be closely related to or strictly defined by substrate (sediment type) and palaeodepth. He claimed that *Chariocrinus andreae* inhabited muddy bottoms and its dense colonies occurred in somewhat deeper waters, below fair-weather wave base, *i.e.,* at depths around 10–15 m (Hess, 1972; Hess, 1999a). In this study, *Chariocrinus andreae* occurs in large amount in both carbonate and siliciclastic rocks. *Balanocrinus subteres* is one of the most cosmopolitan crinoid ever recorded in the Mesozoic and is known from both shallow and deeper environments (compare Table 3 and Fig. 2). Hess (1999a) claimed that another very common Middle Jurassic crinoid taxon, *Isocrinus nicoleti*, frequently occurring in Poland, may also occur within both ooid shoals of the carbonate shelf and in slightly deeper environments (intercalating marls and bioclastic limestones). Tang, Bottjer & Simms (2000) recorded *I. nicoleti* from the shallow-water tidal facies of the Middle Jurassic Carmel Formation in Mount Carmel Junction and also from the open shallow subtidal (carbonate) facies of the Gypsum Spring and Twin Creek Formations of Wyoming (USA). Hunter & Underwood (2009) further noticed that *I. nicoleti* from the Bathonian of England and France, occurred only in shallow-water sediments, represented by low-energy, inner muddy shelf and outer lagoon, oolitic high-energy shoal system, carbonate/muddy low-energy, marine lagoon complex and in the shallow subtidal zone. All these are actually consistent with current observations that indicates *I. nicoleti* (Tables 2 and 3) occurred in both carbonate and siliciclastic rocks, although it preferred shallow-water carbonates. Hunter & Underwood (2009) concluded that the distribution of crinoids corresponds quite nicely to particular facies (but see also the comment on this paper by Salamon, Gorzelak & Zatoń (2010) and reply to it by Hunter & Underwood (2010)). This substrate-dependence is also corroborated by the ANOVA test (although at the generic-level) that the crinoid distribution is highly sensitive for carbonates (limestones) as opposed to siliciclastics (Table 5a–5b).

Of interest is the observation on *Pentacrinites dargniesi*. Hunter & Underwood (2009) also collected *P.* cf. *dargniesi* from shallow, muddy and carbonate habitats. *P. dargniesi*, despite being predominantly found in carbonates, also occurs in offshore claystones of Poland (see Tables 2 and 3). Contextually, it must be keep in mind that *P. dargniesi* was a pseudo-planktonic crinoid that transported on the lower surfaces of driftwood fragments, and hence, is likely to occur in varied facies (see Simms, 1999; Hess, 1999a).

The most numerous isocrinids within the Middle Jurassic of Poland are balanocrinids (*Balanocrinus subteres* and *B. pentagonalis*). Hess (2014a) and Hess (2014b) argued that balanocrinids settled mostly on hardgrounds but they are also be found these in mudstone-dominated settings, possibly on swells of the fine-grained bottom. The largest species of the slender crinoid genus (viz., *B. subteres*) was reported from below storm wave-base (Hess & Spichiger, 2001), where it thrived due to ample nutrient availability and well-oxygenated bottom waters (Hess, 2014a; Hess, 2014b). Klikushin’s (1992) noted, as also in the present study, that *Balanocrrinus subteres* is another most cosmopolitan Mesozoic crinoid species. It occurs in both carbonate and siliciclastic facies and in environments of varying depth, from near shore (close to a beach) to the open shelf, but usually below normal wave base (Table 3 and Fig. 2). This lack of palaeodepth-dependency (although at the generic-level) is also reflected in the ANOVA test that suggests that palaeodepth played no or minor role in the distribution of crinoids (Tables 5c–5d), although very weak (low *p* value) significance is noted for near shore and shallow marine settings, as discussed above, also (see Fig. 2 and Table 5).

On the other hand, Hunter & Underwood (2009) concluded that the occurrences of balanocrinids (*Balanocrinus* cf. *subteres*) were restricted to neritic mudstone facies and brachiopod-rich limestones (*i.e.,* within the offshore, low-energy shelf setting). Hunter & Underwood (2009) also noted that the comatulids show a wide range of distribution from offshore, low-energy shelf up to oolitic high-energy shoal system. In the present study, corroborating previous observations (*e.g.*, (Hunter & Underwood, 2009) (see also Table 5c–5d), the comatulids are restricted to siliciclastic rocks of near shore, below wave-base settings (Table 3 and Fig. 2).

The cyrtocrinids are considered as suggestive of relatively deep-sea depths and with carbonate facies association (*e.g.*, Arendt, 1974; Hess, 1975; Žítt, 1973; Žítt, 1974; Žítt, 1975; Žítt, 1978a; Žítt, 1978b; Žítt, 1978c; Žítt, 1978d; Žítt, 1979a; Žítt, 1979b; Žítt, 1982; Žítt, 1983; Hess, Salamon & Gorzelak, 2011). According to Ausich et al. (1999) and references cited therein), the cyrtocrinids prefer depths exceeding 100 m. Recent cyrtocrinids such as *Cyathidium foresti* live at depths ranging from 380 to 900 m, and *C. plantei* lives at a depth of 200 m (Hess, 1999b). Nevertheless, rare single reports also indicate their presence in shallow (and extremely shallow) environments (*e.g.*, Salamon & Gorzelak, 2007; Salamon, 2019; Krajewski, Ferré & Salamon, 2020). From the Jurassic Yátova Formation of Spain, Zamora et al. (2018) illustrated the spongiolithic facies as having been deposited in relatively shallow and open platform areas with depths not exceeding 60 m, containing the following cyrtocrinid cups: *Eugeniacrinites* sp., *Pilocrinus* sp., *Gammarocrinites* sp.; and a basal circlet of Tetracrinidae indet. that actually belongs to *Tetracrinus moniliformis* (see fig. 9e in Zamora et al., 2018). In the current study, *Gammarocrinites* sp., *Pilocrinus moussoni* and *Tetracrinus moniliformis* occur in carbonates of near shore and shallow marine, to deeper sublittoral under storm influence to open shelf environments.

The roveacrinids represented by the genus *Saccocoma* are exclusive to the shallow marine Bathonian and/or Callovian carbonates of the Tatra Mountains and the Pieniny Klippen Belt (Tethyan province). Kowal-Kasprzyk, Krajewski & Gedl (2020) mentioned saccocomids from the Oxfordian-Kimmeridgian exotic clasts of the Outer Carpathians in southern Poland. Matyszkiewicz (1996) and Matyszkiewicz (1997) mentioned *Saccocoma*-calciturbidites resting on the slope beds of Oxfordian cyanobacterial-sponge carbonate buildups formed in the Polish epicontinental basin (for summary see Fig. 2).

The cyclocrinids are not considered in the present study due to their controversial taxonomic assignment (*e.g.*, Hess, 2008).

Thus, qualitatively (see Fig. 2) isocrinids and cyclocrinids occur in both carbonate and siliciclastic rocks. The cyrtocrinids and roveacrinids occur within carbonate rocks, whereas the comatulids are exclusive to siliciclastics. In terms of palaeodepth, most crinoid groups dominate in shallow environments with the sole exception of cyrtocrinids, that are ubiquitous and occur in both shallow (near shore and shallow marine) and slightly deeper (deeper sublittoral to open shelf) settings. The occurrences of the cosmopolitan taxa, *Chariocrinus andreae* and *Balanocrinus subteres* (isocrinids), is independent of both substrate type and palaeodepth. This qualitative inference (Fig. 2) is largely mirrored quantitatively, also (Table 5).

The quantitative analysis (Fig. 2 and Table 5) suggests that the distribution of crinoids show a strong substrate-dependency (Hypothesis 1) (see Fig. 2 and Table 5) corroborating results by Simms (1989) and Hunter & Underwood (2009). The palaeodepth-dependency (Hypothesis 2) did not yield significant results, although very weak (low *p* value) significance is noted for near shore and shallow marine settings (Table 5), as also reflected in qualitative analysis (see Fig. 2). Although it must be accepted that the quantitative analysis (Table 5) is genus-based. Nevertheless, and until more species-based datasets are available, present quantitative analysis (Table 5) should prove useful in better understanding crinoid diversity trends. These results have important bearing on crinoid diversity studies and underline the importance of incorporating substrate details (facies analysis) while inferring distribution patterns of crinoid species diversity and palaeobiogeography. A more rigorous and quantitative species-based analysis may enable to better understand their varied distribution patterns.

## Conclusion

Almost all Bathonian and Callovian crinoids recorded from Poland dominate in relatively shallow environments. The only exception are cyrtocrinids, that are equally common in both shallow and deeper settings. The most commonly recorded Isocrinids, occur frequently in both carbonate and siliciclastic rocks. Cyrtocrinids also occur likewise, but dominate in carbonates. Roveacrinids are exclusive to carbonates, whereas cyclocrinids occur in both carbonates and siliciclastics. The comatulids occur exclusively in siliciclastics. The most cosmopolitan crinoids are the isocrinids, *Chariocrinus andreae* and *Balanocrinus subtrees*, that occur both in carbonate and siliciclastic rocks, and in shallow and open-shelf environments. Statistical analyses (ANOVA) corroborate these qualitative inferences, and suggests that the distribution of crinoids show strong substrate-dependency (Hypothesis 1) but not palaeodepth-dependency (Hypothesis 2), although statistical significance (albeit very weak; low *p* value) is noted for near shore and shallow marine settings. A more exhaustive species-based analysis may enable to better understand their varied distribution patterns.

##  Supplemental Information

10.7717/peerj.12017/supp-1 Supplemental Information 1 Distribution of Bathonian-Callovian crinoids recorded in Poland within different facies and settings

## Additional Information and Declarations

### Field Study Permissions

The following information was supplied relating to field study approvals (i.e., approving body and any reference numbers): No consent was required as the samples were obtained in natural exposures or in inactive quarries. No consent is needed for this in Poland. There is no institution that could issue it.

### Data Availability

The following information was supplied regarding data availability: All examined specimens are housed in the Institute of Earth Sciences of the University of Silesia in Katowice, Poland, and catalogued under registration numbers: GIUS 8–2510, 8–2569, 8–2571, 8 - 3460Cr, 8-3466, 8-3678/1-6, 8-3734.
