# Supplementary material for: Substrate type and palaeodepth do not affect the Middle Jurassic taxonomic diversity of crinoids
Source: PeerJ. 2021 Sep 13;9:e12017. doi: 10.7717/peerj.12017 (PMC8445083; doi:10.7717/peerj.12017)
Supplement: Supplemental Information 1 [file peerj-09-12017-s001.docx]

**Systematic palaeontology of crinoids recorded in boreholes**

The systematics of crinoids follows the scheme proposed by Hess and Messing (2011). The terminology used herein is from Hess (2006) and Hess and Messing (2011). The stratigraphic ranges are compiled from Hess (1972, 1975), Arendt (1974), Klikushin (1992), Salamon & Zatoń (2006), Salamon (2008a-c, 2009), Hess and Messing (2011), Hess et al. (2011), and Krajewski et al. (2019, 2020).

All crinoids described below come from exclusively from crinoidal limestones (for more details see Table 1).

Order Isocrinida Sieverts-Doreck in Moore, Lalicker and Fischer, 1952

Suborder Isocrinina Sieverts-Doreck in Ubaghs, 1953

Family Isocrinidae Gislén, 1924

Isocrinidae indet.

**Material.** Plenty of partly preserved isolated columnals (both nodals and internodals), brachials, and cirrals. All these remains are also visible in TS and polished slabs.

**Repository.** Laboratory of Palaeontology and Stratigraphy of the University of Silesia in Katowice, Poland, and acronymed with catalogue number: GIUS 8-3734/Iin.

**Remark.** We must keep in mind that brachials and cirrals assigned to Isocrinidae indet. actually may belong to different isocrinid taxa described below. Columnals with residual preserved facets or with invisible facets should also be classified herewith.

**Description.** Columnals are mainly circular, less often (sub-)pentagonal or (sub-)stellate. Nodal height is ca. 120% of the height of an internodal. Nodal diameter varies from 1.2 to 3.8 mm; internodal diameter from 1.5 to 4.0 mm. Cirral scars width varies from 0.25 to 0.9 mm. Petal floors are oval or elongated. Partly visible crenularium consists of moderately thin crenulae. Interradial areas are triangular in shape. Latera may be smooth or covered by ridges. It is just as often straight, concave, or convex. Cirral scars in nodals are circular. Their width ranges from of 18 to 29 % of the nodal diameter and their height averages ca. 65% of the nodal height. Cirrus sockets lie toward the lower margin of the nodal. Distal lip is prominent. A shallow cupule is visible above the cirral scars. Central pore of the cirral scars interrupts a transverse ridge thickened at each end. Lumen of nodals and internodals is predominantly small and circular. Brachials are rather small, V- or U-shaped. Almost all V-shaped brachials have a muscular and syzygial articulation. U-shaped brachials are generally smaller. They display a cryptosyzygy with fine marginal crenulae developed toward the adoral furrow on one side. Cirrals are mainly short and straight.

**Occurrence.** Middle Triassic (Anisian?, Ladinian)-Holocene worldwide.

Subfamily Isocrininae Gislén, 1924

Genus *Isocrinus* von Meyer in Agassiz, 1836

Type species. *Isocrinites pendulus* von Meyer in Agassiz, 1836

*Isocrinus nicoleti* (Desor, 1845)

1845 *Pentacrinus nicoleti* Desor, p. 5, nom. nudum

1975 *Isocrinus nicoleti* (Thurmann); Hess, p. 55, pl. 7, fig.  4, pl. 11, fig. 16

2000 *Isocrinus nicoleti* (Desor); Tang et al., p. 49-50, figs. 4, 5a, b

**Material.** Plenty of partly preserved isolated columnals, 15 almost complete internodals and 2 nodal columnals.

**Repository.** Laboratory of Palaeontology and Stratigraphy of the University of Silesia in Katowice, Poland, and acronymed with catalogue number: GIUS 8-3734/In.

**Description.** Columnals are (sub-)stellate. Crenularium of internodals consists of thin crenulae, up to 28 per petal. Petal floors are distinctly elongated. Nodal articulation is crypto-symplectial with faint crenulae. In both cases adjacent crenulae almost touch each other. Distal facet of internodals is weakly concave while the proximal one is correspondingly convex. Narrow canaliculi on these articula taper inwards to a narrow raised perilumen. Cirrus scars are directed outwards and slightly upwards with only a weakly developed fulcral ridge. Probably two small protuberances are visible at either end. Latera is smooth or slightly convex. Lumen is circular.

For detailed discussion see Salamon & Feldman-Olszewska (2018 and literature cited therein).

**Occurrence.** Middle Jurassic (Bathonian-Callovian) of Europe: Belgium, England, France, Germany, Lithuania, Luxembourg, Switzerland;~~.~~ Asia: Iran; and North America (Bathonian): USA. Poland: Aalenian-Callovian.

Genus *Chariocrinus* Hess, 1972

**Type species.** *Isocrinus andreae* Desor, 1845

*Chariocrinus andreae* (Desor, 1845)

1845 *Chariocrinus andreae* Desor, p. 213

1879 *Cainocrinus andreae* (Desor); de Loriol, p. 112, pl. 14, figs. 31-38

1972 *Chariocrinus andreae* (Desor); Hess, p. 197, figs. 15-20, 22

**Material.** Plenty of partly preserved isolated columnals, 37 complete internodal and 11 nodal columnals.

**Repository.** Laboratory of Palaeontology and Stratigraphy of the University of Silesia in Katowice, Poland, and acronymed with catalogue number: GIUS 8-3734/Ca.

**Description.** Columnals are pentagonal to pentalobate, in some cases they are stellate. Nodals are slightly higher than internodals. Crenularium is well developed and consists of thin crenluae, up to 18 per petal. Petal floors are elongated. Crenulae of neighbouring petal floors nearly touch each other in radial part of the facet. Nodals are higher than internodals and possess five elliptical cirrus scars. Cirri sockets are directed outwards. They are small and have elliptical outline. Latera is smooth. Lumen is small and circular.

For detailed discussion see Salamon & Feldman-Olszewska (2018).

**Occurrence.** Middle Jurassic (Aalenian-Callovian) of Antarctica, Europe and Northern America (Greenland).

Subfamily Balanocrininae Roux, 1981

Genus *Balanocrinus* Agassiz in Desor, 1845

**Type species.** *Pentacrinites subteres* Münster in Goldfuss, 1826-1844

*Balanocrinus subteres* (Münster in Goldfuss, 1826)

1826-1844 *Pentacrinites subteres* Münster in Goldfuss, p. 176, pl. 53, fig. 5

1975 *Balanocrinus subteres* (Münster), Hess, p. 58, pl. 7, figs. 2, 6, pl. 20, fig. 8, pl. 24, figs. 2-3

**Material.** Plenty of partly preserved isolated columnals, 45 complete internodal and 17 nodal columnals.

**Repository.** Laboratory of Palaeontology and Stratigraphy of the University of Silesia in Katowice, Poland, and acronymed with catalogue number: GIUS 8-3734/Bs.

**Description.** Columnals are mainly circular, much less often (sub-)pentagonal. Nodals are distinctly wider and higher that internodals. Petal floors are triangular. Every petal floor is separated by two parallel sets of minute tubercles. Marginal crenulae increase in length toward the radial areas. Adradial crenulae of the adjacent petal floors are fused. Most of the columnals have smooth and straight latera; however, some specimens with slightly convex and knobby latera are also present. Cirrus scars of nodals have an aboral lip. They are directed upwards. Lumen is small and circular.

For detailed discussion see Hess (2014a, b).

**Occurrence.** Early Jurassic? - Late Jurassic of Europe, Africa, Asia, and Northern America.

*Balanocrinus pentagonalis* (Goldfuss, 1826-1844)

1826-1844. *Pentacrinites pentagonalis* Goldfuss, p. 175

2006. *Balanocrinus hessi* Salamon & Zatoń, p. 4-6, fig. 3

**Material.** 21 almost complete internodal and 7 nodal columnals.

**Repository.** Laboratory of Palaeontology and Stratigraphy of the University of Silesia in Katowice, Poland, and acronymed with catalogue number: GIUS 8-3734/Bp.

**Description.** Internodals are pentagonal; proximal columnals are pentastellate. Nodals are pentalobate to pentagonal in outline with swollen interradii in case of pentalobate in shape specimens. All columnals have more or less sharp terminal edges. Petal floors are elongated and lanceolate. Petal floor is surrounded by up to 8 marginal crenulae. Crenularium consists of max. 18 crenulae per petal. Latera is more or less inflated. Lumen is extremely small and circular.

For detailed discussion see Hess (2014a, b).

**Occurrence.** Early Jurassic?, Middle Jurassic - Late Jurassic of Europe, Africa and Asia.

Suborder Pentacrinitina Gray, 1842

Family Pentacrinitidae Gray, 1842

Genus *Pentacrinites* Blumenbach, 1802-1804

**Type species.** *Pentacrinis lapis* Agricola, emended by Bather, 1898 [*P. fossilis* by monotypy =*P. britannicus* von Schlotheim =*P. briareus* Miller]

*Pentacrinites dargniesi* Terquem & Jourdy, 1869

1869 *Pentacrinus dargniesi* Terquem & Jourdy, p. 87-89, fig. 23

1975 *Pentacrinites dargniesi* Terquem & Jourdy; Hess, p. 60, pl. 13, pl. 14, figs. 10-12, pl. 21, fig. 7

**Material.** Plenty of cirrals, 12 almost complete internodals and 2 nodals. Ellipsoidal cirrals are also visible in TS.

**Repository.** Laboratory of Palaeontology and Stratigraphy of the University of Silesia in Katowice, Poland, and acronymed with catalogue number: GIUS 8-3734/Pd.

**Description.** Columnals are pentagonal to (penta-)stellate. Petal floors are long and very narrow. Crenulation is very faintly. Every columnal possesses a unique perilumen surrounding large and circular lumen. It consists of 10, rather thick and raised, beams that build the characteristic mound around the lumen. Central part of nodals possesses symmetric depressions for fixation with internodal. The latera of internodals is smooth, rarely uneven or ornamented with rounded longitudinal carinae. Nodals have 10 cirrus scars that are slightly directed outwards. Cirrals are ellipsoidal in outline. Cirral articular surface sometimes is covered with a distinct fulcral ridge.

For detailed discussion see Głuchowski (1987) and Hess & Messing (2011).

**Occurrence.** Middle Jurassic (Bajocian-Bathonian) of Europe: France, Switzerland. Poland: Aalenian-Callovian.

Order Cyrtocrinida Sieverts-Doreck, in Moore, Lalicker and Fischer, 1952

Cyrtocrinida indet.

**Materials.** Plenty of cup remains, partly preserved columnals and 27 complete columnals.

**Repository.** Laboratory of Palaeontology and Stratigraphy of the University of Silesia in Katowice, Poland, and acronymed with catalogue number: GIUS 8-3734/Cin.

**Description.** Three radials are similar in overall shape and size, but vary in thickness and width. They are wider than high. All lower edges are sharp. Aboral sides are straight and articular facets are narrow. Aboral ligaments are poorly visible. Also transverse ridges are invisible. Axial canals are small and circular. Interarticular ligaments are well developed. Muscle fields are shallow. Few partly more or less complete primibrachials and secundibrachials are non-axillary brachials. They are thick and rectangular. All these plates vary in height and may be curved. Muscle fields encroach on adoral side. Synostosial distal facets are rather circular. Columnals are of various size. Some of them are large; they are tall or short. Others are barrel-shaped, tall and slender to shorter and thicker. Some distal columnals are long and stick-like. Facets are covered by faintly visible crenulae; max. 18 crenulae may be long or short. They are occasionally arranged radially. Lumen may be of different size; sometimes it is small and circular, sometimes large, circular or elliptical. Latera is straight or convex; in some cases it is covered by granules. Columnals mostly large, tall or short. Latera convex or smooth. Articular facets may be covered by 10–16 large, short crenulae.

**Remarks.** Columnals are highly diverse, suggesting that they belong to several different taxa. Circular, barrel-shaped columnals with facet covered by up to 12 long, thick crenulae, and latera covered by tubercles, may belong to sclerocrinids. Others are elliptical in section, tall and slender, sometimes proximally curved; they belong probably to eugeniacrinitids. This suggests that the cythocrinid assemblage from the boreholes of eastern Poland is represented by at least 3-4 taxa (including *Phyllocrinus* sp.). Borehole Żebrak IG 1 is an exception here, from which Salamon & Feldman-Olszewska (2018) described 1 almost complete columnal classified as Cyrtocrinida indet. It was accompanied by several fragmentarily preserved columnals. However, we should keep in mind that these specimens are from a small sample that had undergone destructive maceration.

**Occurrence.** Early Jurassic (Sinemurian)-Holocene worldwide.

Family Phyllocrinidae Jaekel, 1907

Genus ***Phyllocrinus*** d’Orbigny, 1850, in 1850-1852

**Type species.** *Phyllocrinus malbosianus* d’Orbigny, 1850, in 1850-1852

*Phyllocrinus*sp.

**Material.** 2 cups.

**Repository.** Laboratory of Palaeontology and Stratigraphy of the University of Silesia in Katowice, Poland, and acronymed with catalogue number: GIUS 8-3734/Psp.

**Description.** Cup is rosette in shape. Radial cavity is circular, moderately wide and shallow. Cup is narrower at lower part, and gradually expanding up to radial facets. Suture lines between radials are distinct. Facet to stem is large and circular.

**Occurrence.** Early Jurassic?, Middle Jurassic (Bajocian)-Early Cretaceous (Barremian) of Europe: Albania, Austria, Czech Republic, England, France, Hungary, Italy, Portugal, Romania, Russia, Spain, Switzerland, Slovakia, and Ukraine. Poland: Bajocian-Berriasian. According to Hess & Messing (2011) phyllocrinids are also present in Australia and Oceania (New Zealand), although these authors did not specify the age of the sediments in which crinoids would be present. Neither Rasmussen (1961) nor Arendt (1974) in their respective monographs indicated the presence of phyllocrinids in New Zealand.

**Other echinoderms**

Other echinoderms are represented by numerous preserved fragmentarily cidaroid spines, less numerous cidaroid interambulacral plates and asteroid marginal plates. Spines are of a cidaroid type due to presence of small central medulla and wide region of lamellae that are visible in cross-sections. They are represented mainly by clavate elements with longitudinal striation and fairly narrow to strong widened shaft. Spine surface may be ornamented by small and circular to coarse granules arranged longitudinal. These remains are accompanied by cylindrical spines with straight shaft and cortex ornamented by relics of thorns. They are covered by a single primary tubercle with rather large imperforate mamelon. Asteroid plates include both inframarginal and supramarginal plates. Inframarginal plates are ornamented by circular and poorly impressed granule pits and rather large circular tubercles aligned along the proximal edge of the external surface. Supramarginal plates possess shallow lateral fascioles and elevated external surface. Plate surfaces are covered by circular, sometimes polygonal granule pits.

**References**

Arendt, Y.A., 1974. Morskie lilii. Tsirtokrinidy (Sea lilies, Cyrtocrinids). Trudy Paleontologicheskego Instituta, Akademia Nauk SSSR 144, 251 pp.

Agassiz, J.L.R., 1836. Prodrome d’une Monographie des Radiaires ou Échinodermes. Mémoires de la Société des Sciences Naturelles de Neuchâtel 1, 168–199.

Arendt, Y.A., 1974. Morskie lilii. Tsirtokrinidy (Sea lilies, Cyrtocrinids). Trudy Paleontologicheskego Instituta, Akademia Nauk SSSR 144, 251 pp.

Bather, F.A., 1898. *Pentacrinus*, a name and its history. Natural Sciences, 12, 245–256.

Blumenbach, I.F., 1804. Abbildungen naturhistorischer Gegenstände. 7, 36 pp. Göttingen.

Desor, E., 1845. Résumé des études sur les crinoïdes fossiles de la Suisse. Bulletin de la Société neuchâteloise des Sciences naturelles 1, 211–222.

Gislén, T., 1924. Echinoderm Studies. Zoologiska Bidrag Uppsala 9, 330 pp.

Głuchowski, E., 1987. Jurassic and early Cretaceous Articulate Crinoidea from the Pieniny Klippen belt and the Tatra Mts, Poland. Studia Geologica Polonica 94, 6–102.

Goldfuss, G.A., 1826–1844. Petrefacta Germaniae. Abbildungen und Beschreibungen der Petrefacten Deutschlands und der Angränzenden Länder, unter Mitwirkung des Herrn Grafen Georg zu Münster, herausgegeben von August Goldfuss. Band 1 (1826–1833), Divisio prima. Zoophytorum reliquiae, 1–114; Divisio secunda. Radiariorum reliquiae, 115–221 [Echinodermata]; Divisio tertia. Annulatorium reliquiae. I. Bivalvia: p. 65–286; II. Brachiopoda: p. 287–303; vol. 3 (1941–1844); Divisia quinta. Molluscorum gasteropodum reliquiae: p. 1–121; atlas, pl. 1–199. Arnz and Co., Düsseldorf.

Gray, J.E., 1842. Synopsis of the contents of the British Musuem. 44th Edition. iv + 308 pp. London.

Hess, H., 2006. Crinoids (Echinodermata) from the Lower Jurassic (Upper Pliensbachian) of Arzo, southern Switzerland. Schweizerische Paläontologische Abhandlungen 126, 1–144.

Hess, H., 2014a. *Balanocrinus* and other crinoids from Late Jurassic mudstones of France and Switzerland. Swiss Journal of Palaeontology 133, 47–75.

Hess, H., 2014b. *Balanocrinus* (Crinoidea) from the Jurassic: species concept, reconstruction, ontogeny, taphonomy and ecology. Swiss Journal of Palaeontology 133, 35–45.

Hess, H., Messing, C.G., 2011. Treatise on Invertebrate Paleontology, part T, Echinodermata 2, Crinoidea 3. The University of Kansas, Paleontological Institute, Lawrence, 261 pp.

Hess, H., Salamon, M.A., Gorzelak, P., 2011. Late Jurassic-Early Cretaceous (Tithonian-Berriasian) cyrtocrinids from south-eastern Poland. Neues Jahrbuch für Geologie und Paläontologie, Abhandlungen 260, 119–128.

Jaekel, O., 1907. Über die Kӧrperform der Holopocriniten. Neues Jahrbuch für Geologie und Paläontologie, Festband 1907, 272–309.

Klikushin, V.G., 1992. Iskopaemye morskie lilii pentakrinidy i ikh rasprostranenie v SSSR. Leningrad Palaeontological Laboratory, Sankt Petersburg, 358 pp.

Krajewski, M., Olchowy, P., Salamon, M.A., 2019. Late Jurassic (Kimmeridgian) sea lilies (Crinoidea) from central Poland (Łódź Depression). [Annales de Paléontologie](https://www.sciencedirect.com/science/journal/07533969) 105, 63–73.

Krajewski, M., Ferré, B., Salamon, M., 2020. Cyrtocrinids (Cyrtocrinida, Crinoidea) and other associated crinoids from the Jurassic (Kimmeridgian–Tithonian)–Cretaceous (Berriasian–Barremian) of the Carpathian Foredeep basement (western Ukraine). Geobios, 60, 61~~–~~77.

Loriol, P. de, 1877-1879. Monographie des crinoïdes fossiles de la Suisse. Mémoires de la Société Paléontologique Suisse, 4, 1–52; 5, 3–124; 6, 125–300.

Moore, R., Lalicker, C.G., Fischer, A.G., 1952. Invertebrate fossils. McGraw-Hill, New York, Toronto, London, 766 pp.

d’Orbigny, A.D., 1850-1852. Prodrôme de paléontologie stratigraphique universelle des animaux mollusques et rayonnés faisant suite au cours élémentaire de paléontologie et de géologie stratigraphique, vol. 1 (1849 [1850]), 394 pp.; vol. 2 (1850 [1852]), 427 pp.; vol. 3 (1852), 196 pp. + table alphabétique et synonymique des genres et des espèces, 1–189. Masson, Paris.

Rasmussen, H.W., 1961. A Monograph on the Cretaceous Crinoidea. Kongelige Danske Videnskaberne Selskab, Biologiske Skrifter 12, 428 pp.

Roux, M., 1981. Échinodermes: Crinoïdes Isocrinidae. Résultats des campagnes Musorstom 1, Philippines. Mémoires Orstom 91, 477–543.

Salamon, M.A., 2008a. The Callovian (Middle Jurassic) crinoids from the black clays of the Łuków area, eastern Poland. Neues Jahrbuch für Geologie und Paläontologie, Abhandlungen 247, 133~~–~~146.

Salamon, M.A., 2008b. Jurassic cyrtocrinids (Cyrtocrinida, Crinoidea) from extra-Carpathian Poland. Palaeontographica Abt. A 285, 77–99.

Salamon, M.A., 2008c. The Callovian (Middle Jurassic) crinoids from northern Lithuania. [Paläontologische Zeitschrift](https://link.springer.com/journal/12542) 82, 269~~–~~278.

Salamon, M.A., Feldman-Olszewska, A., 2018. Crinoids (Crinoidea, Echinodermata) from the Middle Jurassic (Callovian) of eastern Poland: A case study from the Żebrak IG1 Borehole. Annales Societatis Geologorum Poloniae 88, 273–283.

Salamon, M.A., Zatoń, M., 2006. *Balanocrinus hessi*, a new crinoid (Echinodermata) from the Callovian (Middle Jurassic) of southern Poland. Neues Jahrbuch für Geologie und Paläontologie, Abhandlungen 240, 1–17.

Tang, C.M., Bottjer, D.J., Simms, M.J., 2000. Stalked crinoids from a Jurassic deposit in western North America. Lethaia, 33: 46–54.

Terquem, O., Jourdy, E., 1869. Monographie de l‘étage Bathonien dans le départment de la Moselle. Mémoire de la Société Géologique de France, Paris, 2, 9, 1–175.

Ubaghs, G., 1953. Sous-Classe 4. Articulata J. S. Miller. In: Piveteau, J. (Ed.), Traité de Paléontologie, 3. Masson, Paris, p. 756–765.
